# Ubiquitination and deubiquitination of MCL1 in cancer: deciphering chemoresistance mechanisms and providing potential therapeutic options

**DOI:** 10.1038/s41419-020-02760-y

**Published:** 2020-07-22

**Authors:** Xiaowei Wu, Qingyu Luo, Zhihua Liu

**Affiliations:** State Key Laboratory of Molecular Oncology, National Cancer Center/National Clinical Research Center for Cancer/Cancer Hospital, Chinese Academy of Medical Sciences and Peking Union Medical College, 100021 Beijing, China

**Keywords:** Cancer therapeutic resistance, Apoptosis

## Abstract

MCL1 is an important antiapoptotic member of the BCL-2 family that is distinguishable from other family members based on its relatively short half-life. Emerging studies have revealed the crucial role of MCL1 in the chemoresistance of cancer cells. The antiapoptotic function of MCL1 makes it a popular therapeutic target, although specific inhibitors have begun to emerge only recently. Notably, emerging studies have reported that several E3 ligases and deubiquitinases modulate MCL1 stability, providing an alternate means of targeting MCL1 activity. In addition, the emergence and development of proteolysis-targeting chimeras, the function of which is based on ubiquitination-mediated degradation, has shown great potential. In this review, we provide an overview of the studies investigating the ubiquitination and deubiquitination of MCL1, summarize the latest evidence regarding the development of therapeutic strategies targeting MCL1 in cancer treatment, and discuss the promising future of targeting MCL1 via the ubiquitin–proteasome system in clinical practice.

**Facts**

1. MCL1 is an important antiapoptotic member of the BCL-2 family, and the elevation of MCL1 protein level leads to chemoresistance and correlates with poor prognosis of cancer patients.

2. MCL1 is an unstable protein, and its stability is regulated by the ubiquitin–proteasome system (UPS).

3. Targeting MCL1 appears to be a promising strategy in cancer therapy, but the development of an effective inhibitor targeting MCL1 has begun only recently.

4. Targeting deubiquitinases upstream of MCL1 provides an alternative strategy for inhibiting MCL1 activity.

**Open questions**

1. What are the distinctions and relationships of the various E3 ligases/deubiquitinases that modulate MCL1?

2. Is there a need to identify other E3 ligases or deubiquitinases that modulate MCL1?

3. Can the emerging strategy of targeting deubiquitinases upstream of MCL1 soon be successfully applied in clinical practice?

4. Is the use of a proteolysis-targeting chimera a promising strategy to directly target MCL1 by utilizing the UPS?

## Introduction

Programmed cell death (PCD), a cell suicide process, is essential for normal organ development and tissue homeostasis. However, overactivation or inactivation of the PCD cascade can lead to pathogeneses, such as Parkinson’s disease and tumorigenesis. Apoptosis is the predominant type of PCD, whereas other types have also been discovered in recent years, such as pyroptosis^1^, necroptosis^2^, parthanatos^3^, and ferroptosis^4^. Two distinct pathways can lead to apoptosis, the extrinsic/cell death receptor pathway and the intrinsic/mitochondrial pathway^5^. In the intrinsic pathway, B cell lymphoma 2 (BCL-2) family members have been shown to have a critical role in regulating apoptosis by governing proapoptotic and antiapoptotic intracellular signals^6^. Numerous studies have revealed the subtle regulatory network between BCL-2 family members^7,8^, and their dysregulation can lead to various diseases^9,10^.

Chemotherapy is one of the most widely applied treatments for cancer in humans, and chemoresistance is one of the most severe obstacles that cancer patients face. Apoptosis escape is considered a hallmark of cancer^11^, contributing to the resistance of cancer cells to both chemotherapy^12,13^ and radiotherapy^14^. Due to the regulatory role of the BCL-2 family in apoptotic signaling, studies of the BCL-2 family have elucidated the mechanisms underlying chemoresistance and provided several promising therapeutic strategies. Certainly, emerging attempts to target BCL-2 antiapoptotic members will benefit the treatment of cancer.

## Role of MCL1 in the mitochondrial apoptosis pathway

**Fig. 1:**
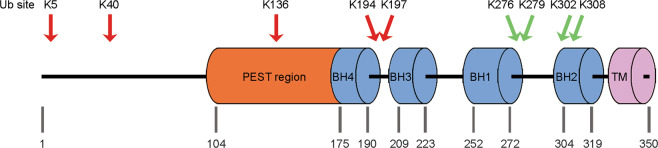
Schematic model of MCL1 protein. MCL1 contains a long N-terminal regulatory domain harboring a PEST sequence, BH1–BH4 motifs and a C-terminal transmembrane (TM) domain. The arrows indicate the sites of ubiquitination (Ub): K48-linked polyubiquitin chains (red) and K63-linked polyubiquitin chains (green).

The BCL­2 family contains more than 25 proteins that regulate the intrinsic apoptosis pathway^15^. Based on their structure and function, BCL-2 family members are classified into three groups: antiapoptotic, proapoptotic, and proapoptotic BH3-only proteins. Antiapoptotic proteins that possess BH1-4 domains include MCL1, BCL-2, BCL-W, BCL-B, BCL-XL, and BFL-1/A1; proapoptotic proteins (apoptosis effectors) harboring BH1-4 domains include BAX and BAK; and proapoptotic BH3-only proteins (apoptosis activators) include BIM, BID, BAD, NOXA, and PUMA^16^. Biochemical and structural studies have revealed that the BH1, BH2, and BH3 regions of antiapoptotic BCL-2 family proteins are proximal to one another and can form a surface-associated hydrophobic groove, a feature allowing interaction with the BH3 amphipathic helix of BH3-containing proapoptotic proteins and physiological antagonization^15,17^. MCL1 is a crucial antiapoptotic member of the BCL-2 family that was initially identified as an immediate-early gene expressed during the 12-*O*-tetra-decanoylphorbol-13-acetate (TPA)-mediated differentiation of the ML-1 human myeloid leukemia cell line^18^. MCL1 contains two PEST domains, four BH domains, and a C-terminal transmembrane (TM) domain as well as an unusually long N terminus that is not observed in other BCL-2 family members^19^ (Fig. 1). The N terminus of MCL1 harbors numerous regulatory residues responsible for caspase cleavage, phosphorylation, and ubiquitination, and contains a PEST motif, a characteristic sequence of rapidly degraded proteins^20^. In addition, the BH1 domain and C-terminal region from aa 303 to 350 are also important for the rapid degradation of MCL1, contributing to its short half-life together with the PEST sequence^21^. The short MCL1 half-life distinguishes it from many other BCL-2 homologs^22^, and allows cells to rapidly switch between survival and apoptotic states in response to various stress signals. Another characteristic of MCL1 is its distinct tissue distribution and expression patterns compared with those of other BCL-2 family members^23^.

**Fig. 2:**
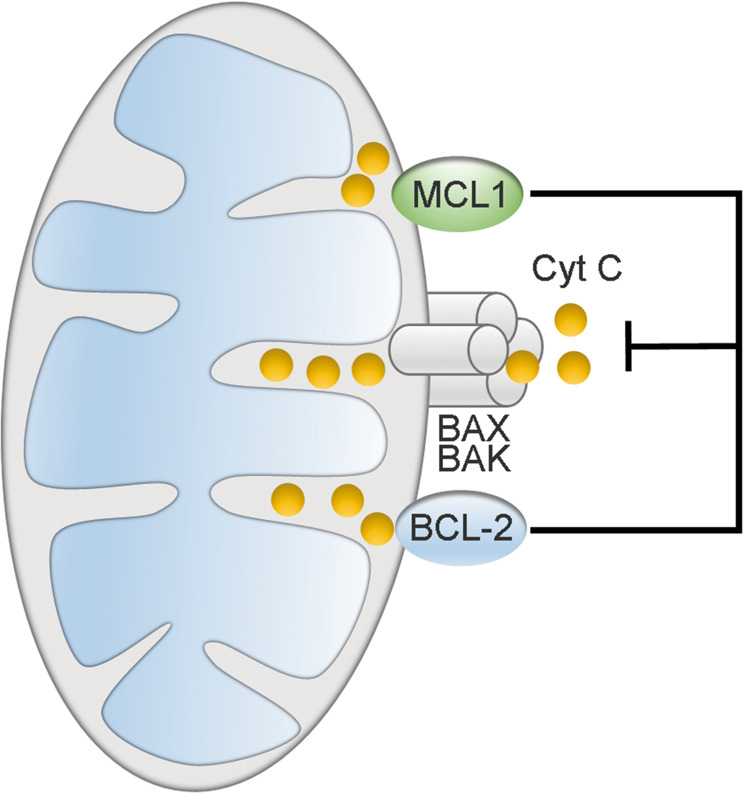
The role of MCL1 in mitochondrial apoptotic signaling. MCL1 interacts with and sequesters proapoptotic proteins to suppress mitochondrial outer membrane permeabilization (MOMP) and cytochrome *c* (Cyt C) release to exert its pro-survival effects.

Several studies have provided a relatively detailed mechanism underlying the antiapoptotic function of MCL1. Classically, the MCL1 protein has been shown to anchor itself to the cytoplasmic face of the mitochondrial outer membrane via its C-terminal TM domain^24^, where it structurally interacts with and sequesters proapoptotic proteins to suppress mitochondrial outer membrane permeabilization (MOMP) and cytochrome *c* release^25,26^ (Fig. 2). Conversely, the above interactions can be antagonized by BH3-only proteins, which displace BAK and BAX from MCL1 to activate the mitochondrial apoptosis pathway^6,27^. In addition to the abovementioned classic antiapoptotic function of MCL1, maintenance of MCL1 levels has been shown to be necessary to preserve mitochondrial morphology and support normal mitochondrial bioenergetic activity in cardiomyocytes^28,29^. In addition, an amino-terminal truncated isoform of MCL1 has been reported to be anchored to the inner mitochondrial membrane (IMM) and exposed to the matrix where it retains the normal IMM structure, mitochondrial fusion, ATP production, membrane potential, and respiration^30^. This mitochondrial matrix form of MCL1 can also directly interact with and modulate very long-chain acyl-CoA dehydrogenase, a key enzyme in the mitochondrial fatty acid β-oxidation pathway, to engage in lipid metabolism^31^. Excluding the functions within mitochondria, MCL1 can be translocated into the nucleus to activate Chk1 and maintain genome integrity in response to DNA damage^32,33^. MCL1 also acts together with BCL-2 and BCL-XL as a transcriptional regulator^34^ or as a stress sensor to participate in autophagy regulation^35^.

## Role of MCL1 in the context of cancer

The amplification and elevated expression of MCL1 has been observed across cancer cell lines and human malignancies. A survey of the expression of antiapoptotic BCL-2 subfamily members in 68 human cancer cell lines revealed that MCL1 mRNA expression was higher than that of other BCL-2 members in lung, prostate, breast, ovarian, renal, and glioma cancer cell lines^36^. Increasing evidence has also shown that MCL1 is highly expressed in multiple cancer subtypes, including hematological malignancies^37^, melanoma^38^, testicular germ cell tumor^39^, hepatocellular carcinoma^40^, breast cancer^22^, urothelial carcinoma^41^ etc. Genetic studies have revealed that the *MCL1* gene is located within 1q21.2, one of the most frequently amplified chromosome regions, and amplified in more than 10% of cancers^42,43^.

Because MCL1 functions as an efficient brake in the mitochondrial apoptosis pathway, it is understandable why MCL1 expression is preferentially increased in cancer cells to sustain their survival in response to various stresses, such as oncogenic stress, X-rays, chemotherapy, and small-molecule inhibitors^44–46^. Indeed, cancer patients with high levels of MCL1 expression have been shown to encounter drug resistance, relapse and poor prognosis outlook. For example, in diffuse large B cell leukemia, AKT-induced aerobic glycolysis promotes MCL1 protein synthesis, thereby maintaining cell survival and resistance to BCL-2 inhibitors^47^. *MCL1* is also frequently upregulated in breast cancer^48^, especially in drug-resistant triple-negative breast cancer (TNBC) after neoadjuvant chemotherapy, with *MCL1* (54%) and *MYC* (35%) gene coamplifications^49^. Elevated MCL1 expression has also been detected in chemoresistant cell lines and patients with ovarian cancer^50,51^.

## MCL1 modulation by UPS

Ubiquitin, a 76-residue polypeptide, is a highly stable and conserved protein^52^, and ubiquitin conjugation is achieved through a cascade of multiple enzymatic reactions catalyzed by three classes of enzymes, including E1 ubiquitin-activating enzymes, E2 ubiquitin-conjugating enzymes, and E3 ubiquitin ligases (E3s)^53^. Eight residues (M1, K6, K11, K27, K29, K33, K48, and K63) within ubiquitin serve as attachment sites for the formation of polyubiquitin chains^54^. Proteins modified with K48-linked chains, the most abundant linkage type in cells, are typically degraded by the 26S proteasome^53^. Non-K48 linkages are primarily involved in nondegradative functions, including cellular signaling, intracellular trafficking, DNA damage response, and chromatin remodeling^52,55^, although their roles are not well defined. The ubiquitination process can be reversed and edited by deubiquitinases (DUBs), which cleave ubiquitin linkages from the substrates to alter their stability or activity^56^. All cellular proteins can be ubiquitinated at least once during their lifetime^57^; therefore, the ubiquitin code permeates every space in the cell and is involved in regulating almost every biological process.

Considering the short half-life and unstable nature of MCL1, posttranslational regulation, especially by the ubiquitin–proteasome system (UPS), is an important mechanism by which high MCL1 expression is maintained in cancer. To date, at least six E3s (Mule, SCF^β-TrCP^, SCF^FBW7^, TRIM17, APC/C^Cdc20^, and FBXO4) have been shown to have a role in the ubiquitination of MCL1^58,59^. The first study suggesting that MCL1 could be regulated by the UPS was published in 2002 but lacked detailed experimental insight^60^. In 2003, two research groups successively reported that the rapid turnover of MCL1 is mediated by the proteasome^61,62^. In 2005, the first molecule shown to directly regulate the ubiquitination of MCL1 was identified through biochemical fractionation of cell extracts^63^. MCL1 ubiquitin ligase E3 (Mule) is a 482-kDa HECT domain-containing ubiquitin ligase that was named for its ability to ubiquitinate MCL1 and induce its degradation in vitro^63,64^. Mule specifically acts on MCL1, as no interactions occur between the BH3 domain of Mule and BCL-XL, BCL-2, or BAX^63^.

The ubiquitination-mediated degradation of MCL1 has been shown to be dependent on its phosphorylation^58^. SCF^β-TrCP^ ubiquitinates and destabilizes MCL1 in a GSK3-dependent manner^65^, whereas the phosphorylation of MCL1 in SCF^FBW7^-induced MCL1 degradation can be mediated by the phosphokinases GSK3, JNK, p38, CKII, or CDK1 depending on the cellular context^66,67^. TRIM17 is the fourth E3 ligase demonstrated to target MCL1^68^. Interestingly, both the ubiquitination of MCL1 by TRIM17 and the association between MCL1 and TRIM17 have also been shown to be associated with the phosphorylation of MCL1 by GSK3^68^. APC/C^Cdc20^ has been shown to engage in the ubiquitination of MCL1 and to control MCL1 stability during mitosis^69,70^; however, existing evidence appears to be insufficient to support the hypothesis that APC/C^Cdc20^ is a bona fide MCL1 E3 ligase. FBXO4 is the last MCL1 E3 ligase identified and is a specific F-box protein that promotes MCL1 degradation in lung cancer^59^. Intriguingly, Choi et al.^71^ showed that an E3 ligase, TRAF6, promoted the K63-linked polyubiquitination of MCL1 on the C terminus and prevented its degradation; these results differ from those of previous studies showing that E3 ligases conjugate MCL1 with K48-linked polyubiquitin chains to promote its degradation.

## Deubiquitinases of MCL1

**Fig. 3:**
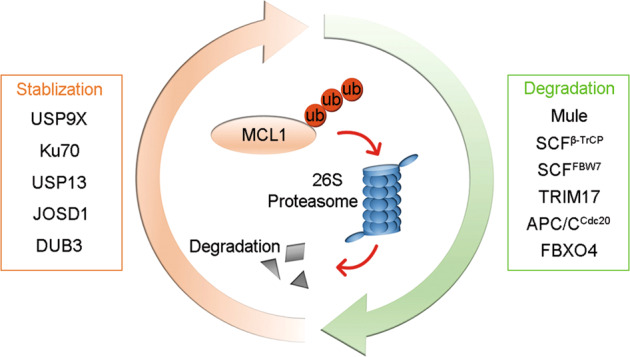
E3 ligases and deubiquitinases balance MCL1 stability. E3 ligases (Mule, SCF^β-TrCP^, SCF^FBW7^, TRIM17, APC/C^Cdc20^, and FBXO4) promote the ubiquitination and degradation of MCL1, whereas deubiquitinases (USP9X, Ku70, USP13, JOSD1, and DUB3) deubiquitinate MCL1 to prevent its degradation.

The discovery of ubiquitination events and E3s related to the critical antiapoptotic protein MCL1 revealed one aspect of its posttranslational modulation. Deubiquitination is the reverse process of ubiquitination, and the ubiquitination of MCL1 can be counteracted by specific DUBs. Therefore, the identification of DUBs regulating MCL1 revealed another side of this modulation process (Fig. 3).

### USP9X

The first DUB targeting MCL1, USP9X, was identified among the immunoprecipitants interacting with MCL1^72^. USP9X prolongs the half-life of MCL1 in an enzymatic activity-dependent manner by specifically cleaving the degradative K48-linked chains on MCL1 to prevent its proteasomal degradation^72^. Structurally, USP9X interacts with the N terminus of MCL1 but not with the BH domain, and the USP9X/MCL1 interaction is relatively weak compared with that between MCL1 and other BCL-2 members, such as NOXA^72^, indicating that this interaction may be easily disrupted. Indeed, subsequent studies showed that NOXA overexpression triggers a decrease in the USP9X/MCL1 interaction, leading to increased ubiquitination and degradation of MCL1 and the induction of apoptosis^73,74^. Schwickart et al.^72^ also showed that maintaining the MCL1 residues Ser155, Ser159, and Thr163 in an unphosphorylated state is crucial for the interaction of USP9X and MCL1. Conversely, MCL1 phosphorylation at these sites by GSK3β disrupts the USP9X/MCL1 interaction, triggers its recognition by E3 ligases and promotes rapid degradation. It appears that phosphokinases regulate the state of MCL1 to determine whether it is degraded by its E3 ligases or stabilized by USP9X. Interestingly, USP9X exhibits biased expression in the brain and immune system^75^, and its function is dependent on the cancer subtype context. USP9X acts as an oncogene in multiple myeloma, lymphoma and non-small cell lung cancer^72,74^ but acts as a tumor suppressor in pancreatic ductal adenocarcinoma and colorectal cancer^76,77^. Moreover, USP9X has also been reported to suppress colorectal cancer progression by stabilizing SCF^FBW7^, an MCL1 E3 ligase^77^.

### Ku70

Lupus Ku autoantigen p70 (Ku70), a DNA double-strand break (DSB) repair protein, has been shown to have an important role in the nonhomologous end-joining (NHEJ) pathway^78^. Intriguingly, in addition to its familiar function, Ku70 has been reported to possess intrinsic deubiquitination activity, cleaving degradative K48-linked polyubiquitin chains from MCL1 and promoting its stabilization^79^. The role of Ku70 as a DUB has been further confirmed by evidence showing that Ku70 directly deubiquitinates MCL1 in a dose-dependent manner and hydrolyzes polyubiquitin chains into monoubiquitin units in vitro^79^. Ku70 specifically stabilizes MCL1 but none of the other antiapoptotic BCL-2 family members^79^. Interestingly, the N- and C termini of Ku70 have distinct functions as the N terminus (aa 1–535) exhibits DSB repair activity, and the C terminus (aa 536–609) is required for the deubiquitination of MCL1 and the antiapoptotic activity of Ku70^79^. In addition, IR enhances MCL1 nuclear translocation, increases the Ku70/MCL1 interaction and reduces the USP9X/MCL1 interaction in a dose-dependent manner^79^. This compensation strategy may be part of the mechanism underlying radioresistance in lung cancer.

### USP13

USP13 was identified as a bone fide MCL1 DUB based on its ability to directly remove the ubiquitin chains modified on MCL1 in vitro^80^. Zhang et al.^80^ showed that both *USP13* and *MCL1* are genomically amplified in numerous cancer types and reported a positive correlation between USP13 and MCL1 in ovarian cancer patient tissues. However, a significant positive correlation between USP13 and MCL1 is not observable in lung cancer patient tissues^80^, and our data even showed a negative correlation between USP13 and MCL1 in nine ovarian cancer cell lines^51^. Previous studies have also shown that USP13 can deubiquitinate essential tumor suppressors (Vps34 complexes, p53, and PTEN) to function as a tumor suppressor in ovarian, breast and bladder cancers^81–83^. Thus, the significance of USP13-MCL1 regulation in the detailed context of cancer must be validated, and the possibility of targeting USP13 as a cancer treatment option must be explored.

### JOSD1

JOSD1 was initially observed to exhibit membrane-related functions^84^, and a subsequent study revealed its antiviral effects via modulation of SOCS1^85^. We performed an in vivo screen to identify essential DUBs that may contribute to the acquired chemoresistance of gynecological cancer, and the results revealed JOSD1 to be a key antiapoptotic DUB whose accumulation leads to acquired chemoresistance in both cervical and ovarian cancer. Our following studies confirmed that JOSD1 acts as a bona fide DUB that cleaves K48-linked polyubiquitin chains on MCL1 and protects it from UPS-mediated degradation. Interestingly, previous studies primarily revealed the role of JOSD1 on the cell membrane and proposed that the deubiquitination activity of JOSD1 is dependent on its monoubiquitination status^84^. In our in vitro deubiquitination assay, we observed that JOSD1 functioned as an MCL1 DUB, and that its deubiquitination activity was independent of its monoubiquitination^86^. Moreover, the colocalization of JOSD1 and MCL1 in the cytoplasm indicates that the DUB activity of JOSD1 may be more important in the cytoplasm than on the membrane, although relatively high JOSD1 expression has been observed on the membrane. In future studies, the JOSD1–MCL1 regulatory axis should be validated in other cancer types.

### DUB3

As mentioned above, each previously reported MCL1 DUB has a largely context-specific effect on MCL1 stability. Furthermore, Zhang et al.^80^ also indicated that several DUBs may affect the stability of MCL1, although they selected USP13 for further studies due to its high amplification rates. Therefore, we performed an unbiased DUB screening to identify the strongest regulator of MCL1. DUB3 was confirmed to be the predominant factor that regulates the stability of MCL1^51^. By analyzing the expression levels of MCL1 and the other DUBs in nine ovarian cancer cell lines, we observed that only DUB3, and not USP9X or USP13, showed a significant positive correlation with MCL1, further supporting our hypothesis that DUB3 is the crucial DUB that modulates MCL1 stability^51^. Moreover, since DUB3 is a veritable oncogene due to its ability to stabilize Snail, Geminin, Cdc25A, NRF2, BRD4, and Cyclin A^87–93^, targeting DUB3 may be a promising strategy in cancer treatment with potentially fewer side effects.

## Targeting MCL1 in cancer treatment

**Fig. 4:**
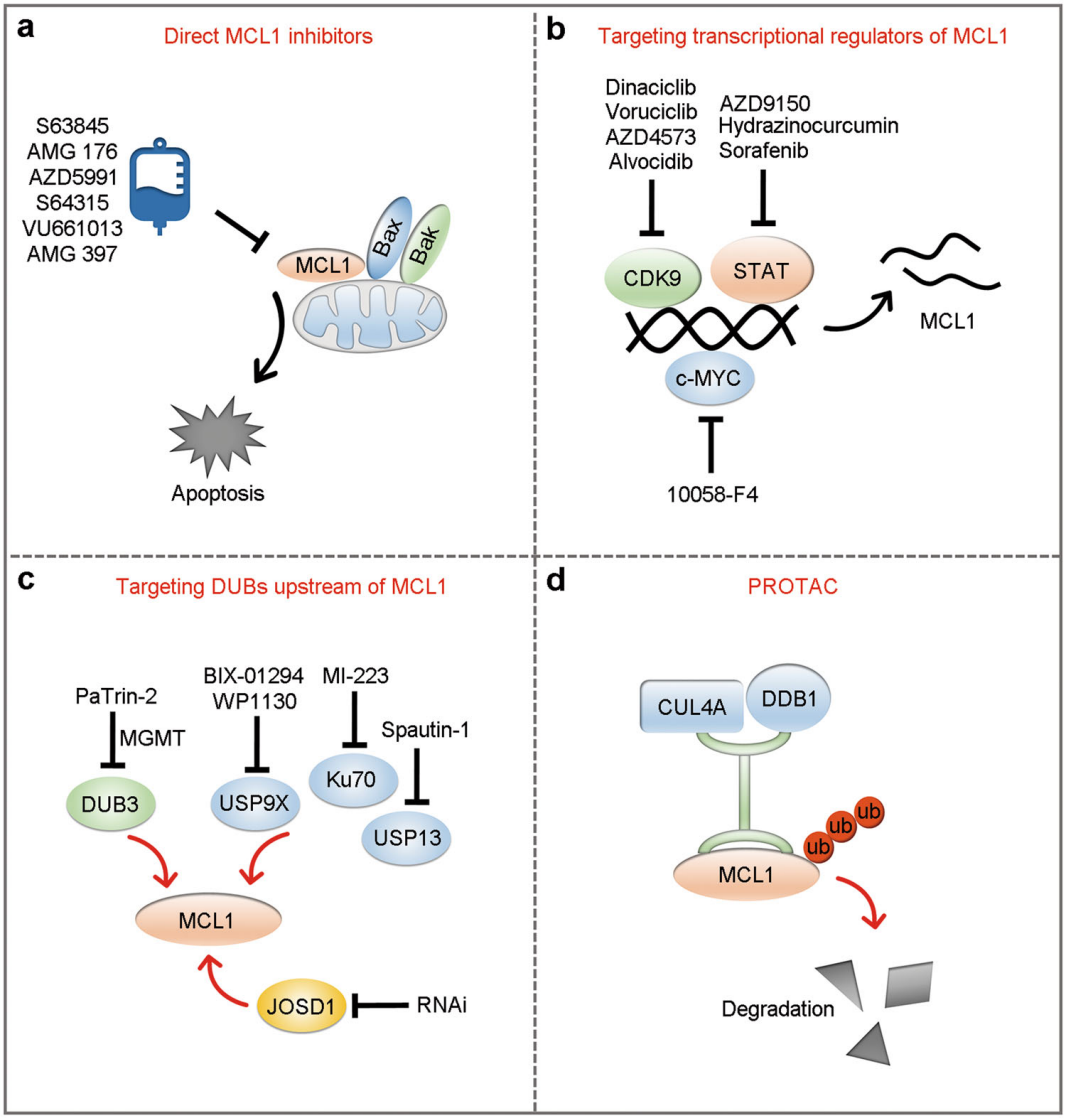
Strategies for targeting MCL1. **a** Inhibitors specifically bind to MCL1, preventing its binding and inhibition of BAX and BAK, and leading to activation of mitochondrial apoptotic signaling. **b** Inhibitors indirectly suppress MCL1 by targeting transcriptional regulators of MCL1. **c** Inhibitors or RNAi indirectly suppress MCL1 by targeting MCL1 DUBs. **d** PROTAC strategy via recruitment of the CUL4A–DDB1 complex to facilitate ubiquitination-mediated degradation of MCL1 protein.

The specific structure of MCL1 had rendered the design of specific MCL1 inhibitors difficult^94^. Fortunately, the emergence of some specific MCL1 inhibitors holds promise for interfering with MCL1, and some BH3 mimetics targeting MCL1 are already being investigated in clinical practice^95–98^. Targeting upstream transcriptional regulators and DUBs of MCL1 has provided another approach to target MCL1. Proteolysis-targeting chimeras (PROTACs) also show a promising future for targeting MCL1 (Fig. 4).

### Development of direct MCL1 inhibitors

As we introduced above, BH3-only proteins displace BAK and BAX from MCL1 to induce apoptosis; thus, drugs mimicking BH3-only proteins might overcome chemoresistance. Conformational analyses showed that the hydrophobic groove of MCL1 is more rigid than that of other BCL-2 family members, and that MCL1 requires a high affinity for its endogenous ligands, which renders the design of MCL1 inhibitors challenging and explains why less progress has been achieved in their development compared with inhibitors targeting other BCL-2 family members^6^. A-1210477 is the first bona fide BH3 mimetic that has been demonstrated to target MCL1, as it selectively binds to MCL1 and disrupts the MCL1–BIM interaction^99^. In 2016, Kotschy et al.^95^ developed the first specific MCL1 inhibitor, S63845, which showed a 20-fold higher affinity than the previously reported A-1210477. The effect of S63845 was shown to be ideal for hematological tumors in subsequent studies^100,101^, although the effects on solid tumors were less pronounced^51,95^. Subsequently, AMG 176, a first-in-class orally bioavailable inhibitor of MCL1, was reported^96^. AMG 176 has two primary advantages over S63845: it can be administered orally and is effective against B cell, monocytic, and neutrophilic leukemia. Soon after the development of AMG 176, another MCL1 inhibitor named VU661013 was also reported^98^.

The sudden emergence of effective MCL1 inhibitors has broadened the horizon for strategies used to target MCL1, and studies have also confirmed the efficacy of using MCL1 inhibitors in combination with other BCL-2 family inhibitors^98,102–104^. Several MCL1 inhibitors, including S63845^95^, AMG 176^96^, AZD5991^97^, S64315^105^, and AMG 397 (https://clinicaltrials.gov), are already being investigated in the clinical stage, and the future of their application in the treatment of chemoresistance is encouraging. Notably, current MCL1 inhibitors show a higher affinity for human MCL1 than mouse MCL1, leading to complications for testing MCL1 inhibitors in pre-clinical mouse models. Fortunately, the recent generation of a humanized MCL1 mouse model provides a solution to more accurately evaluate the tolerability and efficacy of new MCL1 inhibitors in pre-clinical animal experiments^106^.

### Targeting transcriptional regulators of MCL1

As discussed above, the rigid structure and rapid turnover of MCL1 make the design of specific inhibitors against this protein difficult. Therefore, before the recent success of the development of specific MCL1 inhibitors, targeting the regulation of MCL1 was considered an easier strategy than developing an effective BH3 mimetic^107^. One alternative strategy to target MCL1 is by facilitating its transcriptional repression. CDK9 has an important role in regulating gene transcription and has become a potential therapeutic target in cancer treatment^108,109^. Inhibition of CDK9 triggers the suppression of short-lived transcripts and proteins, such as MCL1, to induce tumor cell death^110,111^, and is thus used in concert with BH3 mimetic drugs for cancer treatment^112^. Currently, the selective CDK9 inhibitors dinaciclib, voruciclib, AZD4573 and alvocidib are already under clinical development for the treatment of hematological malignancies^110,111,113^. Another category of MCL1 transcriptional regulator comprises the signal transducer and activator of transcription (STAT) transcription factors. Activation of either STAT1^114^ or STAT3^115^ can promote MCL1 transcription. FMS-like tyrosine kinase-3 signaling also activates MCL1 expression through its STAT5-docking domains^116^. Consistently, both suppression of STAT3 by an antisense oligonucleotide inhibitor (AZD9150)^117^ and inhibition of STAT3 phosphorylation by hydrazinocurcumin^118^ lead to decreased MCL1 transcription. Combined usage of sorafenib (a multikinase inhibitor that suppresses STAT3 phosphorylation) and ABT-737 also synergize to induce glioma cell apoptosis by the inhibition of both MCL1 and BCL-2^119^. c-MYC controls the transcription of MCL1 directly and also regulates BCL-XL by regulating the *eIF4E* gene, in which context the inhibition of c-MYC sensitizes gastric cancer cells to the histone deacetylase inhibitor (HDACi) suberoylanilide hydroxamic acid^120^.

### Targeting DUBs upstream of MCL1

The rapid discovery of several DUBs stabilizing MCL1 has provided another opportunity to indirectly target MCL1 by suppressing DUBs (Table 1). Indeed, USP9X knockdown decreases the level of MCL1 protein, sensitizes MCL1-resistant colon carcinoma and leukemia cells to ABT-737^72^, and sensitizes radioresistant cells to apoptosis induction^121^. The shRNA-mediated silencing of USP9X increases the sensitivity of chronic myelogenous leukemia cells to imatinib and other apoptotic stimuli^122^. BIX-01294, a euchromatic histone–lysine *N*-methyltransferase 2 inhibitor, has been shown to suppress the USP9X-MCL1 axis and induce apoptosis in bladder cancer cells^123^. WP1130, a second-generation tyrphostin derivative, was first identified in screens for AG490-like molecules^124^. Interestingly, WP1130 has also been confirmed to be a small-molecule inhibitor of USP9X activity^125^, and has been shown to trigger apoptosis in myeloid leukemia cells by destabilizing MCL1^122,124,125^.

**Table 1:** Strategies for targeting DUBs upstream of MCL1 in cancer treatment.

DUB	Strategy	Cancer type
USP9X	BIX-01294; WP1130	Hematological malignancies
Ku70	MI-223	Lung cancer
USP13	Spautin-1	Ovarian cancer; lung cancer
JOSD1	AAV-shRNA	Gynecological cancer
DUB3	PaTrin-2	Ovarian cancer

The discovery of Ku70 stabilizing MCL1 in lung cancer is also promising for the development of corresponding inhibitors. Chen et al.^126^ identified a novel small molecule, MI-223, that strongly synergizes with DNA replication stress agents to combat lung cancer by disrupting the MCL1/Ku70 interaction and inhibiting homologous recombination activity. In another comparative study, Wang et al.^79^ showed that compared with that in cells individually treated with Ku70 shRNA or USP9X shRNA, the level of MCL1 in Ku70/USP9X double-knockdown cells was almost undetectable, increasing the sensitivity of lung cells to treatment with staurosporine or the BCL-2 inhibitor ABT-737. Thus, the development of an inhibitor targeting both Ku70 and USP9X, or at least a combination of both Ku70 and USP9X inhibitors, is a feasible approach for further studies. Interestingly, Zhang et al.^80^ showed that pharmacological inhibition of USP13 by a small-molecule inhibitor, spautin-1, markedly downregulated MCL1 protein levels and sensitized ovarian and lung cancer cells to ABT-263, a selective antagonist of BCL-2 and BCL-XL.

We selected DUB3 as an ideal target in ovarian cancer due to its oncogenic functions in various cancer subtypes^51,87–93^. After screening several small-molecule inhibitors, we observed that PaTrin-2 showed the most effective suppression of DUB3 mRNA expression^51^. Mechanistically, PaTrin-2 acts as a pseudosubstrate of O^6^-methylguanine–DNA methyltransferase (MGMT)^127^, and the inhibition of DUB3 transcription may result from the inhibition of its transcription factors by MGMT interference^128^. Functional study results verified that PaTrin-2 effectively reduced the viability of ovarian cancer cells with high MGMT-DUB3-MCL1 expression levels^51^. Most surprisingly, we observed that HDACis could activate DUB3, although the underlying mechanisms remain poorly understood. This “side effect” results in the successful use of PaTrin-2 and HDACis in combination for the treatment of ovarian cancer^51^. Of course, further clinical studies are still necessary to validate this effect. The rapid development of gene therapy has provided flexible methods for modulating the DUBs that stabilize MCL1. However, DUB3 is excluded from this approach because of its high level of homology with several members of the ubiquitin-specific peptidase 17-like family (USP17L1, USP17L3, USP17L4, and USP17L8) since “off-target” effects would be difficult to avoid. For this reason, JOSD1 appears to be the most ideal gene therapy target. Using siRNA-mediated JOSD1 knockdown, we confirmed that the loss of JOSD1 could markedly increase the apoptosis of gynecological cancer cells^86^. Based on this evidence, adeno-associated virus (AAV)-mediated depletion of JOSD1 by shRNA was applied to several ovarian cancer patient-derived xenograft models, and the results showed that AAV treatment targeting JOSD1 significantly suppressed the growth of the xenografts^86^. The results of our study thus provide a promising strategy for the use of therapeutic gene-targeting methods to target DUBs upstream of MCL1.

### Application of PROTACs

The UPS is an evolutionarily conserved apparatus in eukaryotes that is responsible for more than 80% of cellular proteolysis^129^. However, ~80% of proteins in human cells cannot be targeted pharmacologically and are defined as “undruggable”^130^. PROTACs are heterobifunctional molecules capable of bringing a target protein into spatial proximity with an E3 ubiquitin ligase and forming a target-PROTAC-ligase ternary complex to mediate target protein degradation by the UPS^131,132^. The first oral PROTAC drug ARV-110, which targets the androgen receptor for degradation in metastatic castration-resistant prostate cancer patients, was approved by the FDA for phase I clinical trials in 2019^133^. As expected, a recent study developed novel MCL1-targeting PROTACs that effectively bring MCL1 into proximity of the E3 ligase CUL4A–DDB1–CRBN, which labels MCL1 with ubiquitin chains for proteasomal degradation at nanomolar concentrations, resulting in activation of the mitochondrial apoptosis pathway^134^. Importantly, recent studies have shown that BCL-XL PROTAC degraders can direct BCL-XL to CRBN and VHL E3 ligase-mediated degradation while sparing platelets because of the weak expression of CRBN and VHL E3 ligases in human platelets^135,136^. PROTAC technology exhibits advantages of reducing on-target toxicity, which provides insight into minimization of undesirable cardiotoxicity, hepatotoxicity, and hematological toxicity caused by MCL1 depletion via exploration of specific E3 ligases that are rarely expressed in cardiomyocytes, hepatocytes, hematopoietic stem cells, lymphocytes, and neutrophils^28,29,137,138^. These studies have launched the era of the use of PROTACs to target MCL1 in MCL1-dominant cancers, although further supporting studies are urgently needed.

### Limitations for targeting MCL1

Targeting MCL1 has shown great potential in cancer treatment. However, it is noteworthy that MCL1 also has an important role in early embryonic development^139^ and in the survival of multiple normal cell lineages^140^. MCL1 is highly expressed in the myocardium and is crucial for mitochondrial homeostasis and the induction of autophagy in cardiomyocytes^141^. Deficiency of MCL1 in murine hearts leads to rapid and fatal cardiomyopathy^28,29^, and inhibition of MCL1 in human cardiomyocytes results in a severe contractile defect^142^. Furthermore, deletion of MCL1 triggers the loss of hematopoietic stem cells, lymphocytes, and neutrophils^138,143–145^. MCL1 also contributes to the maintenance of hepatic integrity in murine livers^146^, and its absence in murine hepatocytes causes chronic liver damage^137^ and hepatocarcinogenesis^147^. Based on these studies, MCL1 depletion may introduce potential unwanted cardiotoxicity, hepatotoxicity, and hematological toxicity, especially in combination with other cytotoxic drugs. Thus, there is a need to identify a therapeutic window in which cancer cells are more sensitive than normal cells to the loss of MCL1. Interestingly, several groups have found that the loss of a single allele of *MCL1* can kill c-MYC-driven lymphoma cells, an alteration that is well tolerated in normal cells^45,148,149^. This anti-tumor effect while sparing normal cells by partial MCL1 inhibition may allow the establishment of a therapeutic window for MCL1 inhibitors. In addition, improving therapeutic methods to specifically deliver the inhibitors to cancer tissues will be another option to reduce side effects on normal tissues.

## Concluding remarks

Chemoresistance is a severe concern regarding the poor prognosis of cancer patients, and MCL1, an antiapoptotic BCL-2 family member, has become a popular target for cancer treatment due to its important effects. The stalled development of specific MCL1 inhibitors prompted studies of E3 ligases and DUBs modulating MCL1 and the development of their corresponding inhibitors. Considering the rapid turnover of MCL1 and its rigid structure, indirectly targeting MCL1 (e.g., via DUB inhibitors) may represent a promising alternative. Given the number of known MCL1 DUBs and those not yet identified, targeting only one MCL1 DUB seems unlikely to ensure a beneficial effect because the change in MCL1 protein levels may be compensated by other DUBs. Moreover, since DUBs are usually not monospecific for MCL1, off-target toxicity and loss of therapeutic effects are possible in applications targeting DUBs. However, if the dominant DUB that controls MCL1 stability in a specific context can be identified, it would be a promising target. The additional tumor-suppressing substrates of DUB should also be considered. Thus, the reliability of targeting the USP9X-MCL1 and USP13-MCL1 axes should be further explored in specific cancer subtypes. Another potentially large liability of DUB inhibitors is that none have entered the clinical stage except for VLX1570, which targets UCHL5 and USP14, but this clinical trial has been suspended^150^. However, the development of DUB inhibitors is still very young, and numerous DUB inhibitors are in the pre-clinical stage^150^. Therefore, targeting DUBs upstream of MCL1 shows great potential in future research.
